# Comparison of osteogenic capability of 3D-printed bioceramic scaffolds and granules with different porosities for clinical translation

**DOI:** 10.3389/fbioe.2023.1260639

**Published:** 2023-09-28

**Authors:** Xusong Yue, Liben Zhao, Jun Yang, Xiaoyi Jiao, Fanghui Wu, Yan Zhang, Yifan Li, Jiandi Qiu, Xiurong Ke, Xiaoliang Sun, Xianyan Yang, Zhongru Gou, Lei Zhang, Guojing Yang

**Affiliations:** ^1^ Department of Orthopaedics, Rui’an People’s Hospital, The Third Hospital Affiliated to Wenzhou Medical University, Wenzhou, China; ^2^ Bio-Nanomaterials and Regenerative Medicine Research Division, Zhejiang-California International Nanosystem Institute, Zhejiang University, Hangzhou, China; ^3^ Department of Orthopaedics, The First Affiliated Hospital, School of Medicine of Zhejiang University, Hangzhou, China; ^4^ Department of Orthopaedic Surgery, The First Affiliated Hospital of Wenzhou Medical University, Wenzhou, China

**Keywords:** porosity, porous scaffolds, porous granules, osteogenic capability, 3D printing

## Abstract

Pore parameters, structural stability, and filler morphology of artificial implants are key factors influencing the process of bone tissue repair. However, the extent to which each of these factors contributes to bone formation in the preparation of porous bioceramics is currently unclear, with the two often being coupled. Herein, we prepared magnesium-doped wollastonite (Mg-CSi) scaffolds with 57% and 70% porosity (57-S and 70-S) via a 3D printing technique. Meanwhile, the bioceramic granules (57-G and 70-G) with curved pore topography (IWP) were prepared by physically disrupting the 57-S and 70-S scaffolds, respectively, and compared for *in vivo* osteogenesis at 4, 10, and 16 weeks. The pore parameters and the mechanical and biodegradable properties of different porous bioceramics were characterized systematically. The four groups of porous scaffolds and granules were then implanted into a rabbit femoral defect model to evaluate the osteogenic behavior *in vivo*. 2D/3D reconstruction and histological analysis showed that significant bone tissue production was visible in the central zone of porous granule groups at the early stage but bone tissue ingrowth was slower in the porous scaffold groups. The bone tissue regeneration and reconstruction capacity were stronger after 10 weeks, and the porous architecture of the 57-S scaffold was maintained stably at 16 weeks. These experimental results demonstrated that the structure-collapsed porous bioceramic is favorable for early-stage osteoconduction and that the 3D topological scaffolds may provide more structural stability for bone tissue growth for a long-term stage. These findings provide new ideas for the selection of different types of porous bioceramics for clinical bone repair.

## 1 Introduction

Bone, as one of the regenerable tissues in living bodies, involves a series of complex regulatory processes for its development, thus optimizing bone defect repair and functional recovery (Salhotra et al., 2020). Some trauma and disease can cause a certain degree of very large bone defects for the body to make a complete recovery through the self-generated repair mechanisms of bone tissue (Dimitriou et al., 2011; El-Rashidy et al., 2017). Porous implants are expected to treat bone defects by guiding bone tissue ingrowth by providing a building block for cell attachment, proliferation, and maturing into tissue constructs (El-Rashidy et al., 2017; Vijayavenkataraman et al., 2018). Among these, bioceramics have excellent biological performances and structural stability that are compatible with the bone regeneration process and are a promising option for promoting bone repair (Derby, 2012; Shao et al., 2017; Kim et al., 2020; Zhang et al., 2020).

Among a wide range of bioceramics, Ca silicate biomaterials are promising for clinical orthopedics, dental, and craniofacial repair because of their appreciable biocompatibility and osteostimulative properties (Prati and Gandolfi, 2015; Wang et al., 2019; Xu et al., 2022). As for the wollastonite (CaSiO_3_; CSi) ceramic, foreign ion doping into CSi is an effective approach for improving its biodegradation rate and mechanical strength. Our previous studies have demonstrated that when an appropriate amount of Mg atoms occupies the position of Ca atoms in the CSi lattice, the crystal structure of CSi becomes more stable and the Mg ions released can promote the repair of bone tissue, so Mg-doped CSi ceramics (CSi-Mg) could enhance the structural stability and further improve the osteogenic capability (Xie et al., 2016; Ke et al., 2017; Sun et al., 2021; Lin et al., 2022).

The triply periodic minimal surface (TPMS) is an extremely small surface with 3D periodicity. It varies periodically in the triaxial direction, has a smooth surface is completely connected inside the model, and is an excellent porous structure with the advantages of minimum energy and structural stability (Kapfer et al., 2011). The mathematical expressions and construction methods of TPMS have been widely studied, and the properties, such as the shape and stiffness of the structure, can be adjusted by changing the parameters (Hu et al., 2022). Nowadays, TPMS structures have attracted increasing attention because they exhibit highly symmetrical and complex topologies via a computer-assisted design and can be reconstructed by a 3D printing technique (Mustafa et al., 2021; Hu et al., 2022). Based on the high-precision bioceramic stereolithography, the personalized TMPS pore scaffolds have been used to understand the potential bone tissue ingrowth behavior (Kim et al., 2020; Zhang et al., 2020; Lv et al., 2022). Compared to the strut-based porous scaffolds, the TPMS-pore scaffolds have some advantages in studying the angiogenic efficiency and the induction of osteogenic differentiation (Lu et al., 2021; Zhang Q. et al., 2022; Li et al., 2023). It is reported that the high curvature in TPMS pore networks may induce cytoskeleton reorganization, leading to osteogenic and vascular coupling, and accelerated bone regeneration (Yang et al., 2022). Our previous study has found that the curved pore (skeletal IWP) scaffolds exhibit significant flexural strength (≥20 MPa) and that such scaffolds exhibit mild biodegradation and slow loss of mechanical properties *in vitro* (Lu et al., 2021). Hence, it is reasonable to assume that the structural optimization of TPMS pore scaffolds may help understand the bone repair capability for developing implants that match the requirement of critical-sized bone defect repair.

On the other hand, it is well agreed that the optimal implant is to be fabricated as personalized morphology, which may match the bone defect cavity. In general, the conventional large-sized implants lead to another outcome that over-large soft tissue damage is the precondition for the implanting process. In contrast, the granule-type implant is more convenient to fill completely with bone defects. The size and geometry of bone-filling materials are deemed to play a crucial role during bone tissue healing. Indeed, the implant size and porosity could influence the osteoconductive properties and degradation kinetics of the biomaterials (Karageorgiou and Kaplan, 2005; CHOI et al., 2014; Cui et al., 2018; Zhang Y. et al., 2022). Porosity is defined as the percentage of the void space in the solid out of the total volume (León Y León, 1998). Pore parameters and implant morphology can influence bone tissue ingrowth through the modulation of cell behavior and the influence of early angiogenesis (Taniguchi et al., 2016; Bharadwaz and Jayasuriya, 2020; Wu et al., 2022). It is known that bone mineral is an interconnective porous composite including dense and cancellous bone mineral networks, with a dense bone porosity of ∼3.5% and a cancellous bone porosity of 30–95% (Keaveny et al., 2001; Karageorgiou and Kaplan, 2005; Renders et al., 2007). Highly porous implants may significantly improve the osteogenic capability (Wieding et al., 2015), whereas the structural and morphological properties of porous implants may be reduced due to the increased porosity and dimension of the material (Taniguchi et al., 2016). Accordingly, the porous biomaterials should be designed for surgical convenience and structural stability for the bone repair process.

**FIGURE 1:**
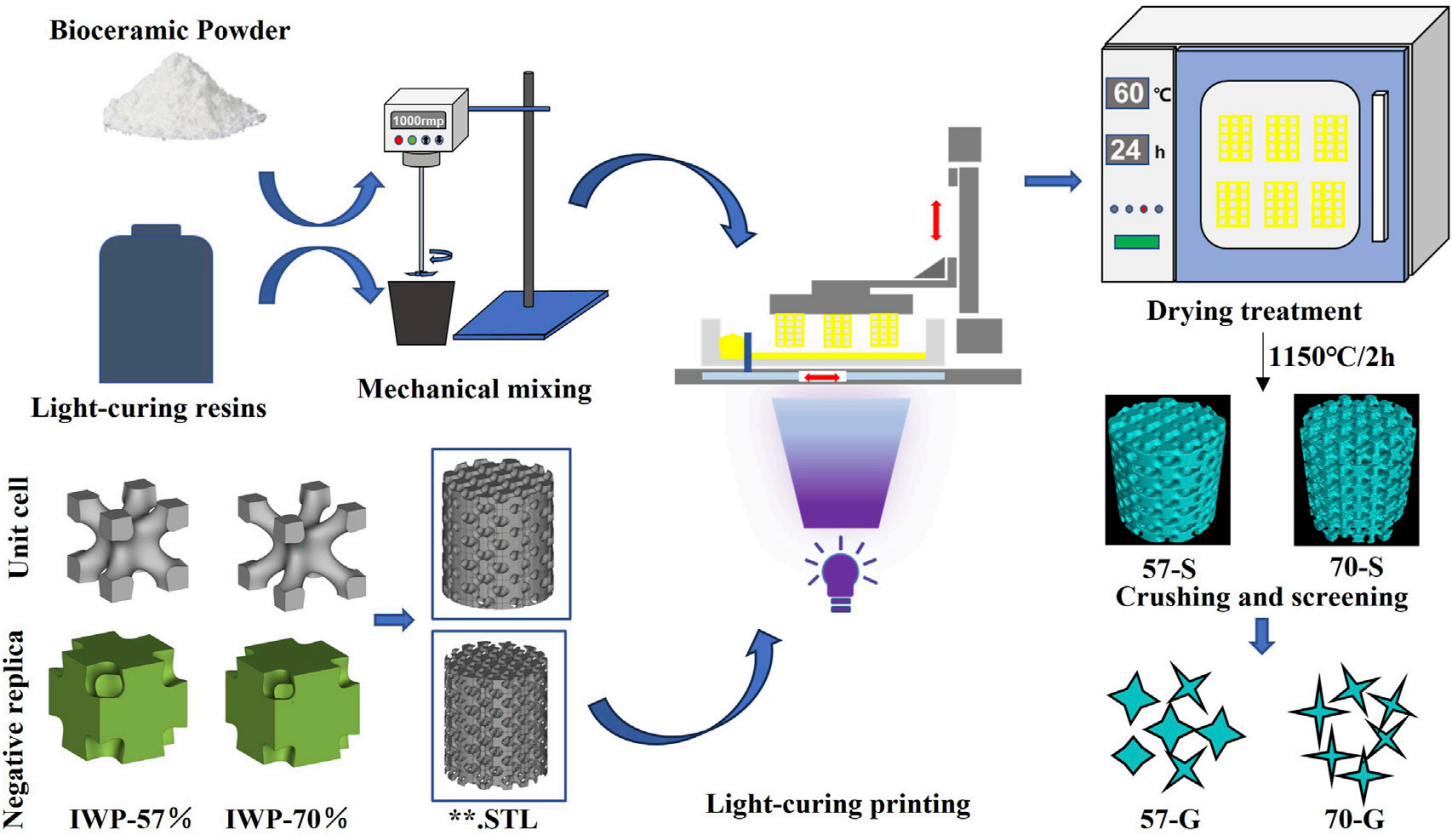
Schematic representation of the process of manufacturing bioceramic scaffolds and bioceramic granules using 3D printing technology.

So far and to the best of our knowledge, there are few studies that focused on the comparison investigation of biomaterial degradation and the osteogenic capability involving porous scaffolds and granules composed of the same chemical compositions. Therefore, the present study aims to evaluate and compare the physicochemical and biological performances of the CSi-Mg5 porous scaffolds and granules *in vitro* and *in vivo*. The two groups of CSi-Mg bioceramic scaffolds with 57% and 70% porosity, respectively, and with IWP cell topology were fabricated by 3D printing technology (Figure 1). The structural parameters of the cylindrical porous scaffolds (∼6.0 mm) and crushed porous granules (∼2 mm) were compared by micro-CT and scanning electron microscopy. The bio-dissolution *in vitro* and osteogenic capability of the scaffolds and granules were evaluated with time in Tris buffer and the rabbit femoral bone defect *in vivo*. The experimental results indicated that both scaffold porosity and morphology significantly affect the new bone regeneration efficiency at the early and late stages, respectively. This will provide clinical guidance for different bone defect conditions and translational biomaterial product design.

## 2 Materials and methods

### 2.1 Chemicals and materials

The reagent-grade inorganic salts (analytic reagent) including calcium nitrate (Ca(NO_3_)_2_∙4H_2_O), magnesium nitrate (Mg(NO_3_)_2_∙6H_2_O), sodium silicate (Na_2_SiO_3_∙9H_2_O), and trishydroxymethylaminomethane (Tris) were purchased from Shanghai Sinopharm Reagent Co., Ltd. and used directly without further purification. The photo-sensitive resin (containing curable monomers or oligomers, photo-initiator, and dispersant) was supplied by TenDimensions Technology Co., Ltd., China. Tris was used to prepare 0.05 M Tris buffer (pH ∼7.40).

### 2.2 Preparation of CSi-Mg5 bioceramic powders

The CSi-Mg5 powders were synthesized by the chemical co-precipitation method, as reported previously (Xie et al., 2016). Briefly, a mixture of the calcium nitrate (0.50 mol/L; Ca(NO_3_)_2_∙4H_2_O) and magnesium nitrate solutions (0.50 mol/L; Mg(NO_3_)_2_∙6H_2_O) was added dropwise to the sodium silicate solution (0.50 mol/L; Na_2_SiO_3_∙9H_2_O) (the molar ratio Ca:Mg:Si = 95:5:100) under the magnetic stirring condition of pH ∼10. After the end of titration, purified water and absolute ethanol were used to wash alternately three times after 12 h of aging. Then, the resulting white precipitate was heated in a 60°C oven for 48 h to fully dry, and the dried powder was calcined at 850°C for 2 h with natural cooling. Finally, the calcined powders were ground in an ethanol medium with a rotation speed of a planetary ball mill (Chisun Technology Co., China) at 300 rpm for 6 h. The powder was dried overnight at 60°C. X-ray diffractometry (XRD; RIGAKU D/max RA) using CuK-*α* radiation was observed at 40 kV/40 ma. Data were collected between 10° and 60° in steps of 0.02°/2θ to identify the crystallization of powder. The contents of Ca, Mg, and Si in the powders were measured by inductively coupled plasma optical emission spectrometry (ICP-OES: 710-es, Varian, United States).

### 2.3 3D model design for printing porous scaffolds

Based on our previous studies, two IWP-type structural cells with different porosities (57% and 70%) were first designed using MathMod (Li et al., 2021) (Figure 1). The average pore size of the unit cell in 3D space was then calculated using Avizo software, and their average pore size was maintained by adjusting their dimensions to ∼550 μm. The designed structural cells were then repaired in detail using Materialise Magics 21.0 software. The designed structural cells and, finally, the two types of cylindrical scaffolds were designed using the periodic 3D filling function of the software application for subsequent experimental studies. The Ø 10 × 10-mm cylindrical scaffold model was designed for the subsequent preparation of porous bioceramic granules, while the Ø 6 × 6-mm cylindrical scaffold model was designed for the *in vitro* determination of biodegradability, mechanical properties, and *in vivo* osteogenic properties, and subsequent implantation in animal experiments.

### 2.4 3D printing of porous ceramic scaffolds and granules

Based on our previous experiments, the volume loading of the CSi-Mg5 powder is about 60% (Lu et al., 2021). The slurry for 3D printing was prepared by mixing 60wt% CSi-Mg5 powder and 40wt% photosensitive resin, and then stirring for 30 min using a high-speed mixer to ensure the two were well mixed (Li et al., 2019). A stereolithography machine (TenDimensions Technology Co., China) with a wavelength of 405 nm was used for photocurable printing of the bioceramic scaffolds. The powder resin paste is poured into the printing tank, and the squeegee pushes out a thin layer of paste on the glass plate. Then, the appropriate exposure parameters were selected, and UV light was irradiated from below the glass plate to form a cured layer of ∼100 μm thickness per layer on the forming table. After printing was completed, the printed samples were placed in deionized water for more than 10 min to ultrasonically remove the residual paste on the surface of the scaffolds, and then placed in a 60°C oven for a period of thorough drying. Finally, the scaffolds were placed in a muffle furnace, heated to 400°C at a rate of 1°C/min, and held for 1 h to ensure complete volatilization of the resin in the scaffolds, and then heated to 1,150°C at a rate of 2°C/min, held for 2 h after the holding period, and naturally cooled to obtain the bioceramic scaffolds. The porous bioceramic scaffolds were crushed into porous granules using a pressure testing machine and sieved through 20-mesh and 10-mesh molecular sieves, to obtain porous ceramic granules with an equivalent volume granule size ranging from 0.85 to 2.00 mm.

### 2.5 Primary morphology and structure analysis

The morphology and pore structure of the porous scaffold and granules were observed using a mobile camera (Nova 6, Huawei). The samples were coated with a thin layer of gold, and the surface and fracture microstructures of the bioceramic scaffolds were examined by scanning electron microscopy (SEM; JEM-6700F; Japan). Linear shrinkage of cylindrical scaffolds before and after sintering was determined using a digital caliper. The ideal model volume was analyzed using Magics 21.0 software to obtain the ideal model volume, and the cylindrical volume was excluded from the model volume to obtain the ideal void volume, and both calculations gave the ideal model porosity.

### 2.6 2D/3D microstructure analysis

Microcomputed tomography (vivaCT 100, Scanco Medical, Switzerland) was used to scan sintered scaffolds (*n* = 3) and pellet scaffolds filled in cylindrical rigid polyvinyl chloride (PVC-U) molds (Ø 10 × 6 mm) at a resolution of 14 μm and an exposure time of 3,000 ms to analyze void percentages and volumes. The 2D/3D pore structure was reconstructed using auxiliary software (Volume Graphics MAX, Volume Graphics, Germany), and the software application (Volume Graphics MAX 3.0.2) was used to calculate quantitative data on pore parameters including mean pore size, porosity, and surface area.

### 2.7 Biological degradation *in vitro* evaluation

Samples were taken from each group for weighing and recording the initial weight (m_0_; *n* = 6), and the scaffolds and granules were immersed in Tris buffer (pH ∼7.40) at a solid–liquid ratio of 1.0 g/50 mL at 37°C. At six time points on days 1, 3, 7, 14, 21, and 28 after immersion, 1 mL of the supernatant was aspirated and supplemented with an equal volume of fresh Tris buffer, and the supernatant was diluted 10 times with deionized water for ICP-OES (Thermo, United States) analysis. The samples were removed after 1, 2, 3, and 4 weeks of immersion, washed with anhydrous ethanol, and then dried at 60°C for 12 h. The weight (m_1_) of each group of samples after immersion was weighed and the weight loss was calculated using the following formula: mass loss = m_1_/m_0_ × 100%.

### 2.8 *In vitro* surface remineralization experiments

First, the porous scaffold samples were immersed in simulated body fluid (SBF) at the ratio of the scaffold surface area/volume of solution of 0.1 cm³/mL. The inorganic ion concentrations were similar to those of human plasma (Na^+^, 142 mM; K^+^, 5 mM; Ca^2+^, 2.5 mM; Mg^2+^, 1.5 mM; SO_4_^2−^, 1 mM; HPO_4_^2−^, 1 mM; Cl^−^, 36 mM; and HCO_3_^−^, 14 mM), and the containers were sealed and placed in a 37°C thermostat. After 7 days of immersion, the samples were dehydrated and dried, and then gold-sprayed for the apatite production observation by SEM. The elements on the surface layer of the samples were qualitatively analyzed by energy dispersive X-ray (EDX) to calculate the Ca/P ratio.

### 2.9 Scaffold implantation and specimen collection

All animal experiments were conducted and handled according to the standards of the Ethics Committee of Zhejiang University. The New Zealand white rabbits (∼3.0 kg) were divided into three groups of 10 rabbits each, and the bioceramic scaffold and granule samples (57-S, 70-S, 57-G, and 70-G) were equally implanted into each group of rabbit footpad models by cross-matching. All New Zealand white rabbits were acclimatized for more than 1 week prior to surgery under steel cage feeding conditions, and food boxes were removed 8 h before surgery. Rabbits were anesthetized with fresh 3% sodium pentobarbital (Merck, Germany) at a dose of approximately 1.0 mL/kg by intravenous injection at the ear margin. After completion of anesthesia, the surgical area of the lateral condyle of the distal femur was shaved and disinfected. A longitudinal skin incision of approximately 3 cm was made in the plane of the lateral femoral condyle, and the fascia was separated layer by layer to reach the bone surface; then, a Ø6 × 6-mm defect was created perpendicular to the bone surface with a dental ring drill, and the autoclaved bioceramic scaffolds and granules were filled into the constructive defect. The wound was then closed layer by layer, and an appropriate amount of penicillin powder was spread before closing the wound to prevent bacterial infection. Postoperatively, the rabbits were left free in the cage, and veterinary penicillin (800,000 units) was administered intramuscularly daily on days 1, 2, and 3, while the postoperative incision was observed for changes. The rabbits were euthanized by injection of an overdose of sodium pentobarbital after 4, 10, and 16 weeks post-implantation, and femoral specimens were collected and placed in test tubes filled with 4% paraformaldehyde fixative for storage.

### 2.10 μCT scanning analysis

Quantitative analysis of the microstructure of animal samples was performed by microelectronic computed tomography (μCT; Inveon, Siemens, Germany). First, the specimens were scanned along the longitudinal axis with a slice thickness of 15 μm at 90 kV and 56 mA current. The data obtained after scanning were passed through Inveon Acquisition Workplace (IAW, Siemens, Germany) software to reconstruct 3D images of the region of interest (ROI, Ø 6.0 × 6.0 mm) containing the repair area. The osteogenic indices BV/TV (ratio of bone volume to the total defect volume), RV/TV (ratio of residual material volume to the total defect volume), and Tb⋅N (number of bone trabeculae) were quantified for the bone defects of the ROI.

### 2.11 Histological and histomorphometric analyses

After radiological examination, the groups of specimens were subjected to histological analysis. The specimens examined were thoroughly cleaned, and then dehydrated and prepared as hard tissue sections by embedding the specimens in polymethyl methacrylate (PMMA). The embedded implant was sliced in a direction perpendicular to the long axis of the femur to a thickness of approximately 100 μm using a slicer (SP1600; Leica). The sections were then slowly ground with a micro-grinder (Exakt-Micro-Grinding System, Leica, Germany) to a thickness of approximately 40–50 μm and polished on a Macintosh machine. The sections were then polished and finally stained with MacNeal and Masson stains, and analyzed by light microscopy (DMLA, Leica; Germany) at different magnifications (×40, ×200). For histomorphometric analysis, ×100 magnification images of the sections were selected and analyzed using Image-Pro Plus 6.0 (Media Cybernetic, United States) image analysis software. The area of the newly formed bone (BS) and the total area (TS) were measured quantitatively, and then, BS/TS was calculated from the acquired data (*n* = 4).

### 2.12 SEM/EDS characterization

The sections were sprayed with gold and analyzed by field emission SEM (FE-SEM; Hitachi, Tokyo, Japan) and energy dispersive X-ray spectrometry (EDS; Apollo X; EDAX, Inc., Mahwah, United States) at an accelerating voltage of 15 kV. The Ca/P atomic ratio was determined, and four regions were randomly selected for analysis.

### 2.13 Statistical analysis

SPSS 21.0 (IBM, United States) was used as the statistical software application for this experiment, with all quantitative data expressed as the mean ± standard deviation. Differences between experimental data were examined by one-way analysis of variance (ANOVA). Differences in results were considered statistically significant when *p* < 0.05.

## 3 Results

### 3.1 Printing technique for the porous scaffolds

The bioceramic frameworks were printed as shown in Figure 1. Quantitative measurements of the bioceramic scaffold size were made on cylindrical samples before and after the sintering treatment (Table 1). The sintering process resulted in linear shrinkage of porous bioceramics, accompanied by higher shrinkage in the *X*–*Y* axis (∼24.49 ± 0.1% and ∼25.35 ± 0.1%) than in the *Z*-axis (∼25.59 ± 1.3% and ∼23.75 ± 1.2%). The high-porosity 70-S samples showed slightly higher shrinkage than the 57-S samples. However, the 57-S samples had a larger specific surface area (4.85 ± 0.1 m^2^/kg) than the 70-S samples (4.30 ± 0.1 m^2^/kg).

**TABLE 1:** Structural parameters for the bioceramic scaffolds (Ø 6 × 6 mm) with a porosity of 57% and 70%.

Sample	Diameter (mm)	Height (mm)	Specific surface area (m^2^/kg)	X–Y shrinkage (%)	Z-shrinkage (%)
57-S	5.89 ± 0.01	5.96 ± 0.1	4.85 ± 0.1	24.49 ± 0.1	23.59 ± 1.3
70-S	5.82 ± 0.01	5.95 ± 0.1	4.30 ± 0.1	25.35 ± 0.1	23.72 ± 1.2

### 3.2 Preliminary characterization of porous bioceramics

According to the XRD analysis for the CSi-Mg5 powder, the diffraction peaks were identified as the wollastonite 2M phase (PDF#43-1460), indicating that Mg doping leads to no phase change in wollastonite (Supplementary Figure S1). Meanwhile, the ICP analysis also indicated that Mg replacing Ca was approximately 4.77%, which is close to the designed value of 5%.

**FIGURE 2:**
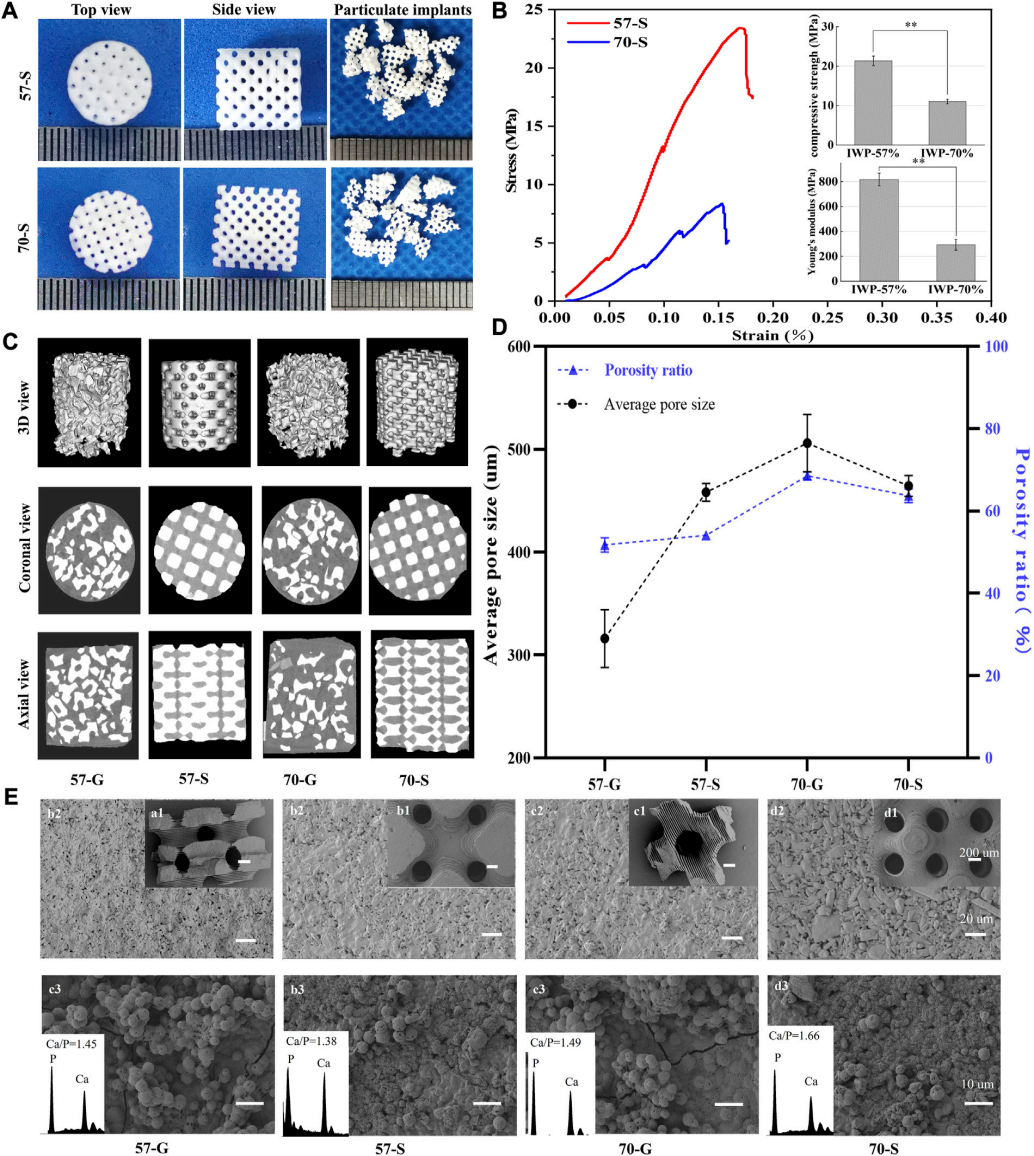
**(A)** Top and side views of 57-S and 70-S bioceramic scaffolds after sintering and appearance of 57-G and 70-G bioceramic granules after sieving the crushed scaffolds; **(B)** stress–strain curves and initial compressive strength and Young’s modulus for 57-S (red) and 70-S (blue),**p < 0.01. **(C)** 57-S and 70-S bioceramic scaffolds, 57-G and 70-G bioceramic granules filled into Ø 6 × 6-mm molds, by CT scan 3D views, and axial and coronal views; **(D)** average pore size and average porosity analysis; **(E)** SEM micrographs of bioceramic scaffolds and granules 50 × (a1, a2, a3, and a4), 500 × (b1, b2, b3, and b4), and after *in vitro* mineralization 1,500 × (c1, c2, c3, and c4).

Figure 2A shows the outward appearance of bioceramic scaffolds with a precisely defined pore geometry after sintering, and the porous granules derived from the structural collapse of the scaffolds were also observed using a digital camera. The cylindrical scaffold and designed pore architectures were maintained well after sintering, except for some shrinkage. In particular, the fully interconnected macropores within the scaffold can be seen from the side-view observation. Figure 2B shows the mechanical behavior of the bioceramic scaffolds (57-S and 70-S) with different porosities under the compression condition. In total, the average compressive strength of 57-S was 21.7 MPa, while that of 70-S was 11.5 MPa, which is nearly half of the former. In comparison, the 57-S scaffold has a higher Young’s modulus value (∼817.06 N/m^2^), indicating greater resistance to deformation. These scaffolds showed an initial approximately linear increase in stress at strains less than 0.15%. When strain reaches 0.154%, the stresses in the high-porosity scaffold reach a high front and brittle separation occurs earlier. In total, two groups of scaffolds showed similar deformation tendencies in the compressive strain (∼0.154% and ∼0.173%).

Meanwhile, the complete porous structures could be reconstructed by μCT, and the 2D/3D pore network and pore morphology were confirmed (Figure 2C). From the cross-sectional view, the 70-S scaffolds showed more interconnected pores than the 57-S samples, and the former has thinner pore wall thickness than the latter, implying that such a 3D printing technique may readily fabricate the high-precision porous constructs with different porosity via adjusting the pore wall dimension. In contrast, the porous granules showed irregular morphology and different sizes, and meanwhile, such granules could hardly achieve the closely packed filling in the defect, although the granule-packed implants have fully interconnective pore architectures. Moreover, the filling density of 57-G groups was higher than that of 70-G, possibly attributed to the lower porosity of the former. The axial and coronal views of the CT images clearly showed the difference in the filling behavior of the scaffold and granules. The 57-S and 70-S scaffolds were highly dense filling in comparison with 57-G and 70-G, with smaller intergaps and less porosity. From the (specific) surface areas of the scaffold and granules by measured μCT, the granules showed higher surface areas than those of the scaffolds, and the 57-G and the 70-G granules had more appreciable surface areas of 15.31 ± 0.88 and 14.15 ± 0.15 cm^2^, respectively.

It was mentioned that the 57-G and 70-G groups showed significantly different porosities (51.72% ± 1.73% *vs.* 68.5% ± 0.94%; Figure 2D). A similar difference occurred between the 57-S and 70-S groups (54.04% ± 0.95% *vs.* 63.70% ± 1.7%; *p* < 0.01). It was worth mentioning that, in total, the 70-G granules had the highest among the four groups of porous bioceramic fillers. Figure 2D shows the pore dimensions of each group measured by CT images. The pore sizes of 57-G and 70-G were 316.21 ± 28.09 and 505.80 ± 27.85 μm, respectively. The mean pore diameters of 57-S and 70-S were 458.53 ± 8.63 and 464.20 ± 10.134 μm, respectively, which were slightly smaller than the theoretical pore size based on the CAD model design.

The pore geometry of bioceramic scaffolds and granules was observed by SEM (Figure 2E). As expected, the regular macropores were retained well after sintering. Moreover, the immersed scaffolds in SBF for 14 days showed a new apatite-like remineralization layer. It can be found that a dense new surface layer was formed onto the pore wall. The quantitative EDS analysis showed that both Ca and P peaks occurred on the surface layer, with surface Ca/P ratios ranging from 1.38 to 1.66. The bioceramic granules could also induce the new apatite-like depositing mineral.

### 3.3 Assessment of bio-dissolution and ion release *in vitro*

**FIGURE 3:**
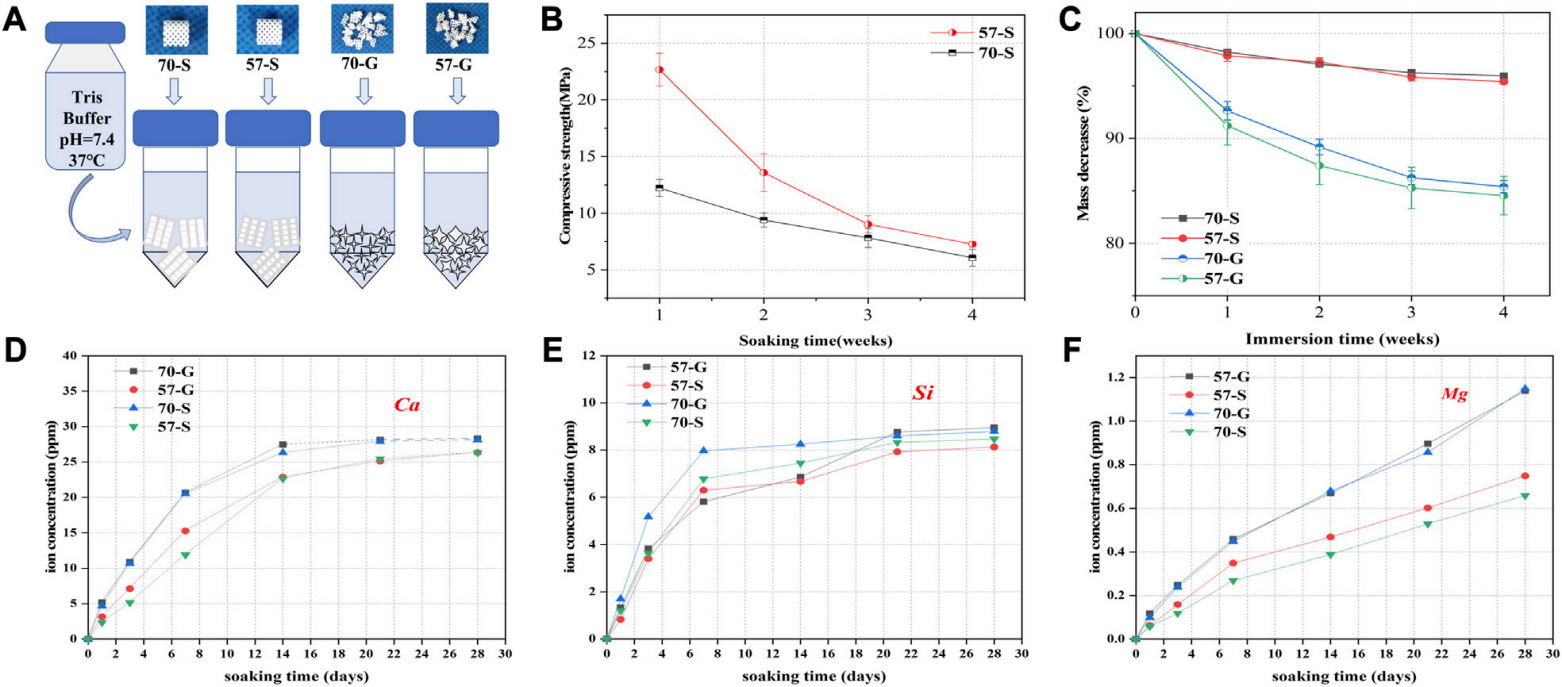
Degradation, mechanical loss, and ion release experiments were performed in Tris buffer (pH = 7.4.37°C). **(A)** Schematic diagram of bioceramic scaffolds and granules immersed in buffer; **(B)** variation in weight (%) of bioceramic scaffolds and pellets *vs.* immersion time; **(C)** variation in stress resistance of bioceramic scaffolds *vs.* immersion time; and **(D–F)** variation in ion concentration *vs.* immersion time.

The bioceramic scaffolds and granules were used to evaluate the *in vitro* biodissolution behavior (Figure 3A). As shown in Figures 3A, B, fast mechanical decay of the scaffolds occurred during the initial 2 weeks of immersion, accompanied by 45% and 18% in decay for the 57-S and 70-S scaffolds, respectively. Indeed, the scaffolds still had appreciable compression resistance (∼11.5 and ∼9.4 MPa). Then, the scaffolds maintained a slower decrease in compressive strength (6–7 MPa) after 4 weeks. All bioceramic porous samples exhibited mass decay in Tris buffer (Figure 3C). During the immersion process, the granular bioceramics showed significantly faster biodissolution in comparison with the porous scaffolds. The scaffolds had a mass loss of only ∼4% after 4 weeks of immersion but that of the granules lost nearly 15% (Figure 3C). It is interesting that the low-porosity samples showed higher mass loss than the high-porosity counterpart, although there was no significant difference (*p* < 0.05).

Figures 3D–F show the changes in Ca, Si, and Mg concentrations of the porous scaffolds and granules in Tris buffer. All samples degraded rapidly within the initial 7 days of immersion, showing a rapid increase in ionic concentrations. In particular, Ca and Mg concentrations showed a steady increase at the end of the immersion test (28 days), while the silicon concentration remained very stable with the prolongation of immersion time after 7 days. It is worth noting that, on the other hand, the porous granules exhibited a higher biodegradation rate than the scaffolds before the structural collapse, possibly due to the higher appreciable specific surface area in the aqueous medium. Obviously, faster ion release implies faster material biodissolution and mass decay, as shown in Figure 3C.

### 3.4 Evaluation of bone regeneration *in vivo*

#### 3.4.1 *In vivo* macroscopic evaluation

**FIGURE 4:**
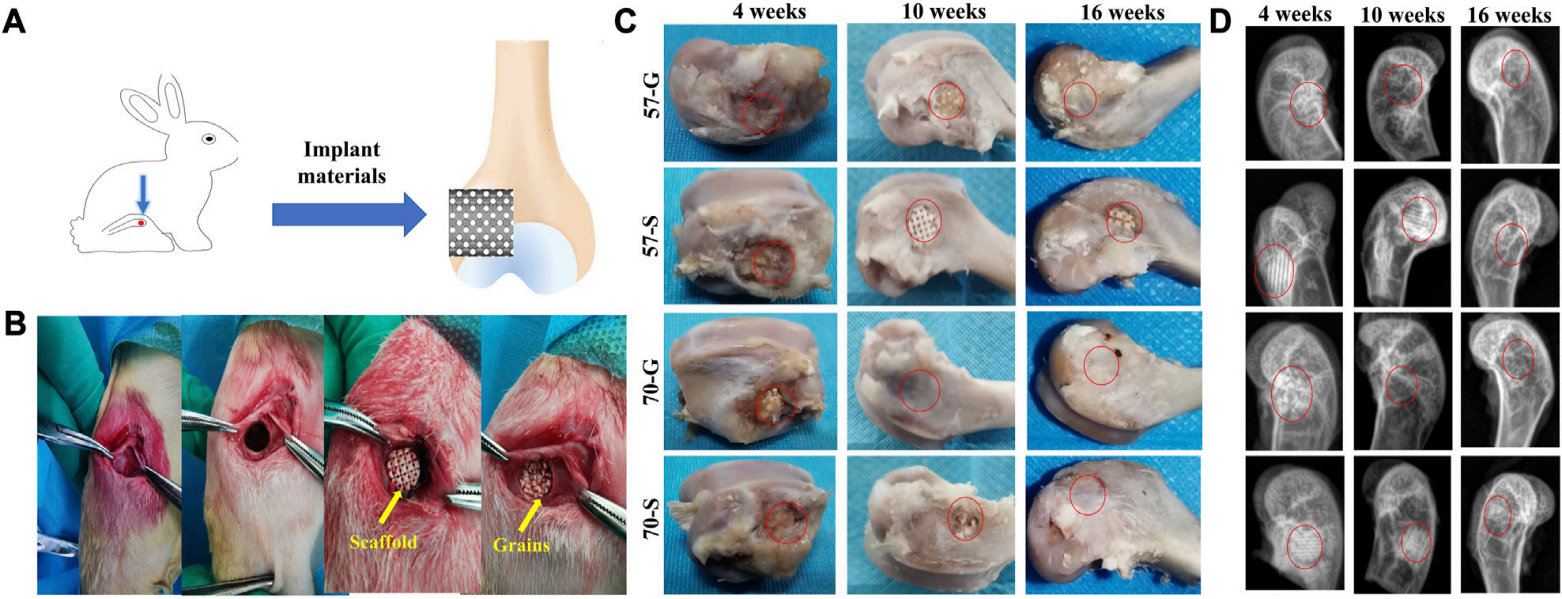
**(A)** Schematic diagram of bioceramic implantation into the femoral condyle of a New Zealand white rabbit; **(B)** bioceramic scaffold and granule implantation procedure; **(C)** bone specimens at 4, 10, and 16 weeks; and **(D)** X-ray images of femoral defects filled with bioceramic scaffolds and granules at 4, 10, and 16 weeks after implantation.

Implantation protocols with porous scaffolds and granules were designed to systematically investigate their effect on bone regeneration efficiency *in vivo*. The rabbit femoral condyle model with defective material implantation is shown in Figure 4A. The implantation procedure is shown in Figure 4B. The rabbits showed no signs of infection after the procedure, moved well, and survived long enough to be harvested. The general view of the femoral condyle specimens is shown in Figure 4C. The area of repair at the defect site became progressively larger over time, and no necrosis was observed in any of the specimens. The porous granules were significantly more effective than the porous scaffolds, according to the significant difference between 70-G and 57-S implants. At 16 weeks, the repair tissue could completely cover the defect surface.

Radiographic examination showed that the porous scaffolds and granules showed variable biological progression from 4 to 16 weeks postoperatively (Figure 4D). The more expected bone repair was observed from the 57-G and 70-G groups. In contrast, the bone defects filled with 57-S were less well repaired at 4 and 10 weeks. At 10 weeks, the 57-S and 70-S scaffolds were easily distinguished from the host bone tissue. In contrast, 57-G and 70-G groups exhibited more rapid *in vivo* biodegradation from 4 to 16 weeks. Interestingly, at 16 weeks, radiographs of the 70-S, 57-G, and 70-G groups showed almost no discernible difference in implanted materials, which implies that the higher porosity of the scaffold and, especially, the granule fillers may contribute to the biodegradation of CSi-Mg5 bioceramics.

#### 3.4.2 μCT *in vivo* examination

**FIGURE 5:**
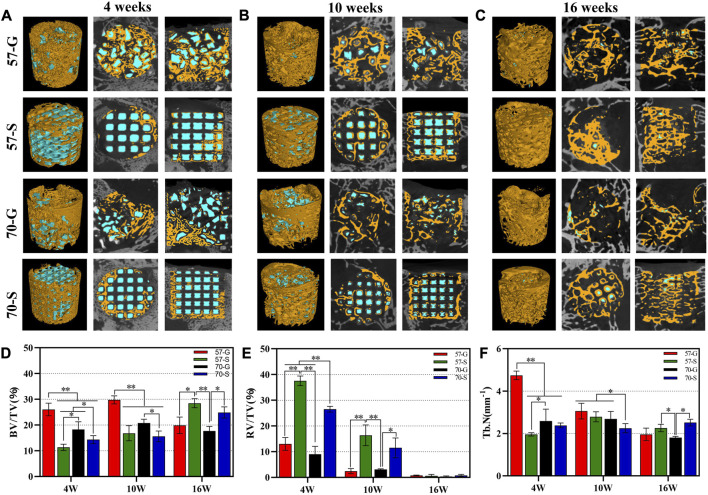
**(A–C)** Two- and three-dimensional μCT reconstructions of femoral defects filled with bioceramic scaffolds and granules were performed 4, 10, and 16 weeks after implantation; new bone (yellow); bioceramic (blue); BV/TV **(D)**, RV/TV **(E)** and Tb.N **(F)** of the bone defects were quantified according to the analysis of the three-dimensional μCT reconstructions. *p < 0.05 and **p < 0.01.

Figures 5A–C show the 2D/3D μCT images of femoral condylar defects at 4–16 weeks postoperatively. All implants showed a porous structure as observed from the similar reconstruction in coronal and sagittal images (the internal pore walls of all implants were rendered as blue by the software application). The 2D μCT examination showed the infiltration of new bone tissue into the granule-filled bone defects at 4 weeks (Figure 5A). Interestingly, the granule-filling groups (57-G and 70-G) showed more appreciable new bone tissue ingrowth than the scaffold groups. The internal gaps between the 57-G granules were all filled with new bone tissue and completely bridged. However, the new bone tissue infiltration was only in the peripheral regions of the bioceramic scaffolds within the initial 4 weeks.

With the prolongation of implantation time up to 10 weeks, significant new bone tissue invasion into the macropore architectures in the 57-S and 70-S scaffolds and new bone tissue had arrived at the internal pores of the scaffolds (Figure 5B). At this time point, there was more osteogenesis in the 57-S group than in the high-porosity 70-S group. Notably, in the 57-G granule group with a large amount of internal bone tissue production, the material residual was significantly reduced.

At a later stage (16 weeks), the 57-S and 70-S groups had a large area of newly formed bone tissue, accompanying a continuous new bone mineral network in the bone defect, similar to the situation observed from the 57-G and 70-G groups (Figure 5C). It was noted that the scaffolds and granules were nearly biodegraded completely from 10 to 16 weeks, leaving only a small amount of isolated islands, but interestingly, the new bone tissue was grown along the interconnected pore networks in the porous scaffolds, without the structural collapse.

Quantitative characterizations, including BV/TV, RV/TV, and Tb.N data, were consistent with the aforementioned observations (Figures 5D–F), and the differences among the four groups of fillers depended only on the implant morphology and porosity. At 4–10 weeks, BV/TV data were higher for the granule groups (57-G and 70-G), reflecting continued bone tissue ingrowth. This also reflected the higher Tb.N data and higher bone repair efficiency. This suggests that granular materials with irregular and disordered pore networks may be significantly beneficial for early-stage bone tissue regeneration. Interestingly, however, the BV/TV data showed a significant decrease in the 57-G and 70-G groups at 10–16 weeks, suggesting the new bone tissue began remodeling during this stage. Obviously, the 57-S and 70-S groups showed a significant increase in the BV/TV data from 10 to 16 weeks, with the scaffold group overtaking the granules group at 16 weeks. Meanwhile, the BV/TV data on the 57-S group outperformed those of the 70-S group, suggesting that the stable pore structure of bioceramic scaffolds with apricate porosity also favors osteoconduction in the long term. On the other hand, during bone tissue maturation and remodeling, a transient decrease in the new bone tissue was quantified in femoral condylar defects. From 4 to 10 weeks, the RV/TV data decreased more rapidly in the porous granule group compared to the scaffold group, and the former biodegraded more rapidly, especially the 70-S scaffold degraded faster than the 57-S scaffold. It was worth noting that at 16 weeks, there was no significant difference in the RV/TV data and the measurements showed almost universal biodegradation in all groups.

#### 3.4.3 Histological evaluation of femoral bone specimens

**FIGURE 6:**
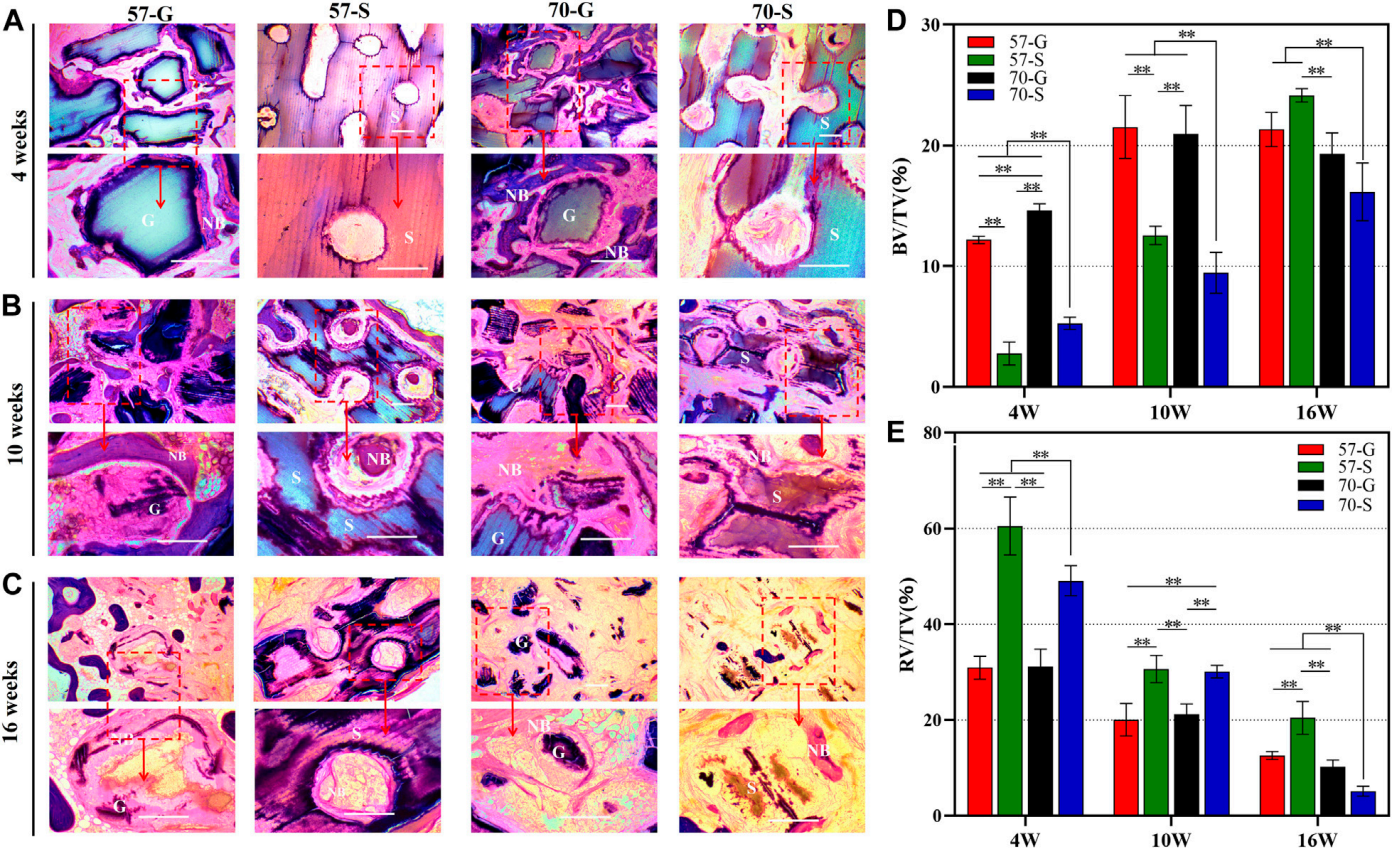
MacNeal-strained photomicrographs (×40, ×100) at 4 weeks **(A)**, 10 weeks **(B)**, and 16 weeks **(C)** of implanted bioceramic scaffolds and granules. The percentage of the new bone area **(D)** and that of the residual material **(E)** were analyzed for each group at different implantation times by histometry. NB, newly formed new bone; G, bioceramic granules; S, bioceramic scaffold. *p < 0.05 and **p < 0.01.

The PMMA-embedded bone specimens were then sectioned at 100 μm, and the implanted material within the circular bone defect and the surrounding neonatal tissue was examined by light microscopy. MacNeal staining was then performed with reagents, and the tissue sections were examined by high-magnification microscopy (40, 100×). As shown in Figure 6, the bioceramic scaffolds and granules are gray, and the new bone tissue and fibers are pink and pale blue, respectively. The MacNeal-stained images showed that at 4 weeks, no necrosis or inflammatory reaction was evident in any group, and the porous network/tissue interface was observed. The new bone tissue formed mainly around the pore wall of the bioceramic scaffold and extended into the interconnected pores. The growth of the new bone tissue into the bone defects varied according to the different implant morphologies. An interconnected pore network with different sizes was formed between the 57-G and 70-G groups. As for the scaffold groups, 57-S and 70-S, the ordered pore networks were undoubtedly facilitated with the growth of new bone tissue ingrowth, whereas only a small amount of new bone tissue was produced in the early stages (Figure 6A).

At 10–16 weeks, MacNeal’s staining showed the histomorphological evolution of the implant/tissue interface (Figures 6B, C). We observed a large amount of bone tissue uniformly distributed within the implant connection holes in all groups (Figure 6B). In both the porous granule and scaffold groups, both the μCT scan sections and histological specimen observation showed that the bone tissue was continuously attached to the pore wall, especially in the 57-G and 70-G groups at 10 weeks. At that time point, the new bone tissue invaded inside the gaps and integrated with the bioceramic granules. However, the scaffold structure was not significantly deformed, with significant bone tissue appearing within the pores and a steady increase in the surrounding bone tissue until 16 weeks. A large area of mature bone tissue surrounded the scaffold, comparable to the 57-G group. On the other hand, the 70-S scaffold showed significant material degradation and structural changes at 16 weeks but no significant difference from the granule groups.

The quantitative analysis of NB in the scaffold using MacNeal-stained sections is shown in Figure 6D. At 4 and 10 weeks, the amount of bone formed in the granule groups was significantly better than that in the scaffold group (*p* < 0.01) and 57-G was better than 70-G (*p* < 0.01). At 16 weeks, the osteogenic volume of 57-S was higher than 70-G and 70-S (*p* < 0.01), possibly due to the faster biodegradation of 70-S (Figure 6E) and the inability to maintain a continuous stable support structure internally. The 57-G and 70-G granules decreased slightly, consistent with the μCT-derived data, possibly because the granules degraded faster throughout the implantation process.

**FIGURE 7:**
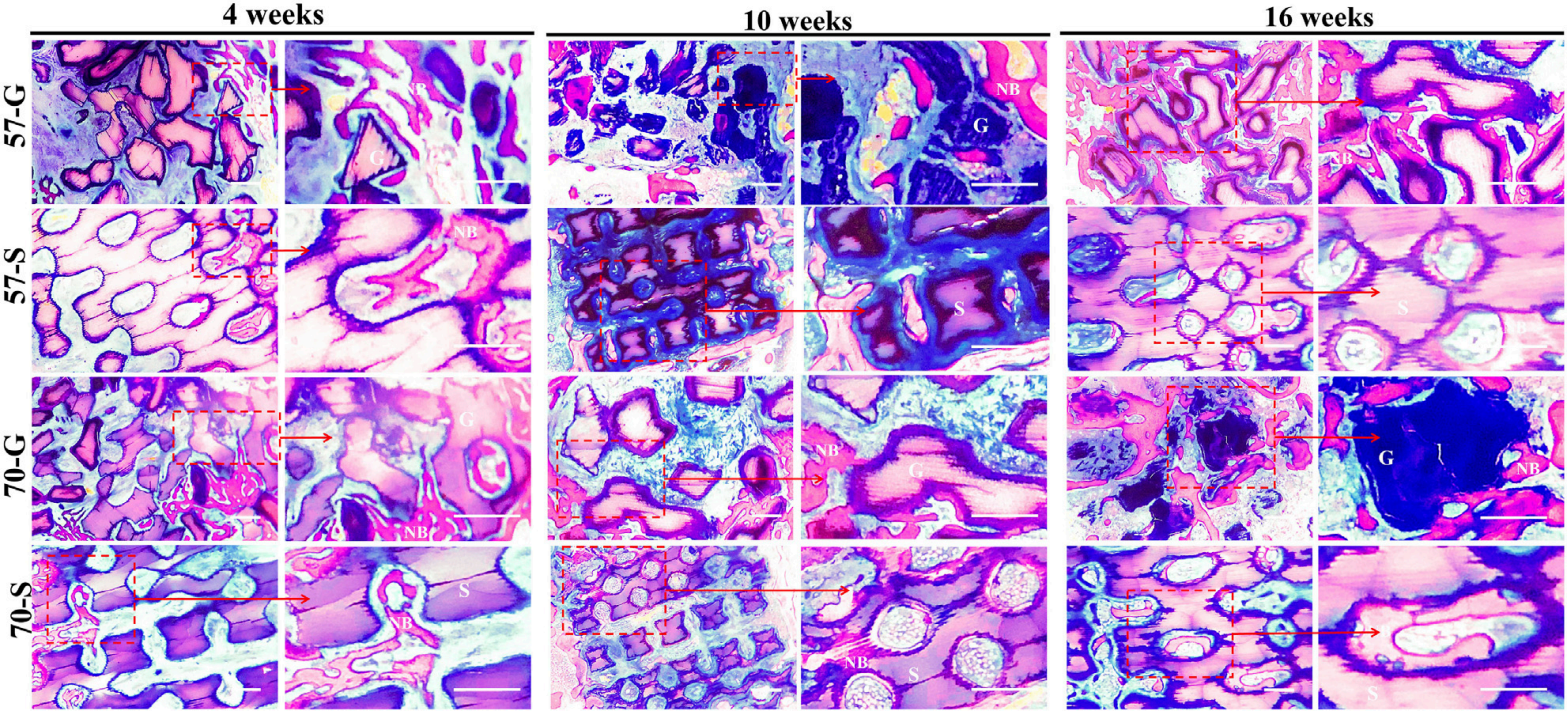
Histological evaluation (Masson staining; ×40, ×100) and the bone regeneration of bioceramic scaffolds and granules at 4,10, and 16 weeks. NB, newly formed new bone; G, bioceramic granules; S, bioceramic scaffold.

The Masson’s stain images also showed the maturation of the nascent bone tissue (Figure 7). At 10 weeks, a large network of collagen fibers (blue) was visible in the 57-S group, while a distinct mature bone tissue (red) was visible in the 57-G and 70-G groups. After 16 weeks, most of the collagen fibers have transformed into mature bone tissues.

#### 3.4.4 Evaluation of the chemical composition of phase transformation

**FIGURE 8:**
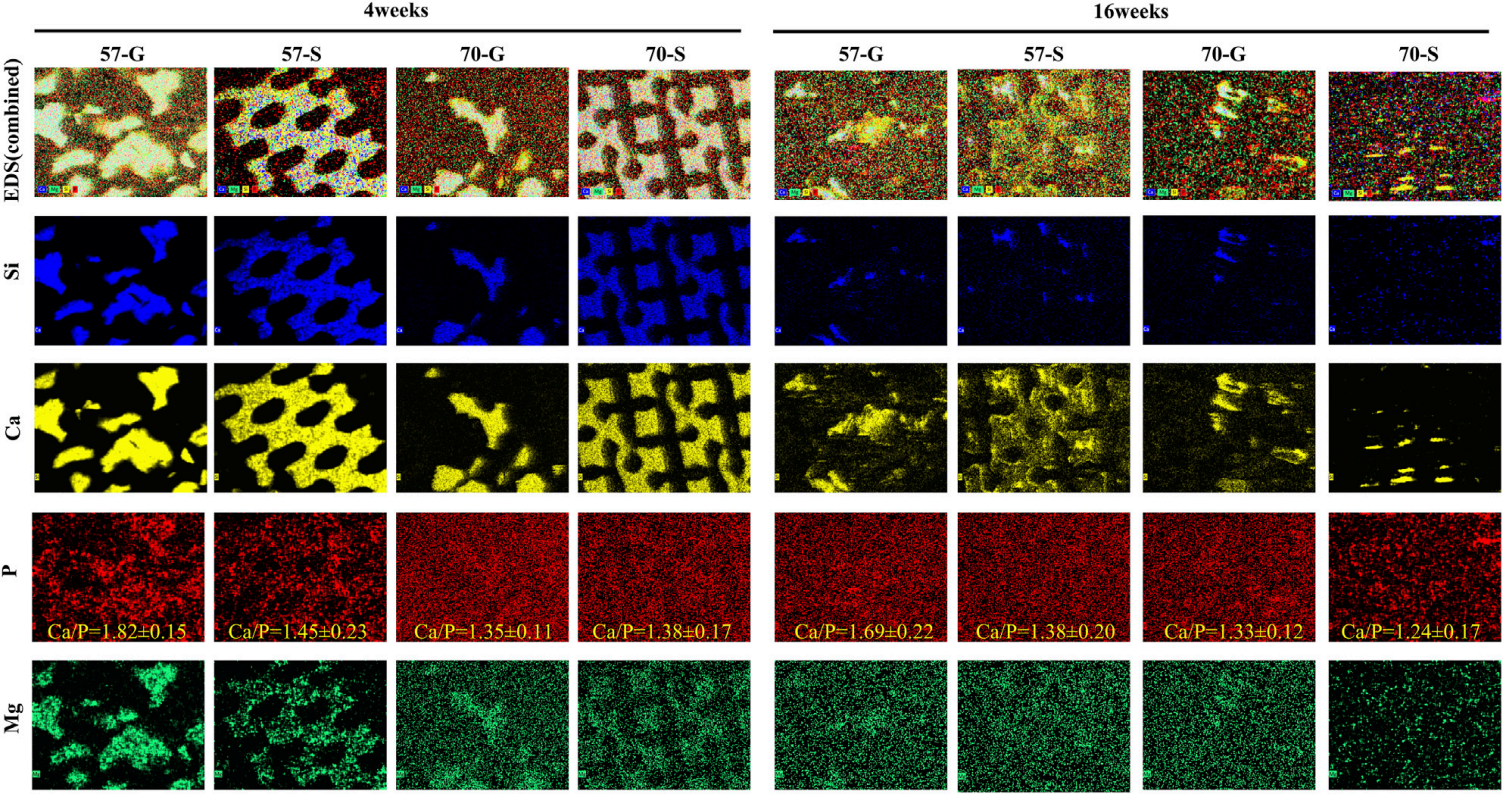
SEM images of the bioceramic struts after 4 weeks **(A)** and 16 weeks **(B)** of implantation and EDS mapping of Si, Ca, P, and Mg (50×). According to EDS mapping, the different components overlap on the SEM images. Mineralized tissue (P-rich) formed around a bioceramic pillar (Si-rich).

In order to evaluate the transformation process and elemental distribution of bioceramic implants to apatite-like bone minerals, SEM/EDS analysis was used to examine the untreated specimen sections at 4 and 16 weeks (Figure 8). From the SEM and EDS mapping images, inorganic ions including Ca, Mg, Si, and P were randomly distrusted using different colors to distinguish. The bioceramic scaffold was rich in Ca, Mg, and Si, and P-rich in areas of the new bone tissue. After 16 weeks, the Ca- and Si-rich areas were further reduced, and P and Mg showed homogeneous distribution. This can be attributed to the biodegradation and phase conversion of the Ca-/Si-rich bioceramics into Ca-/P-rich apatite. In particular, in the 70-S group, the scaffold structures have almost disappeared and P was evenly distributed. The Ca/P ratios in bioceramic regions were shown in the P plot.

## 4 Discussion

Various attempts have been made in bone tissue engineering to repair bone defects by optimizing the preparation of osteoconductive biomaterials, among which the design of porous scaffolds similar to the natural bone mineral network is of great importance for bone tissue regeneration (Yoshikawa et al., 2009; Kim et al., 2017). Among these, porous scaffolds and granules have been used in filling various bone defects; however, in clinical practice, the size and shape of the bone defect cavities often vary and are irregular. The customized bioceramic scaffolds tend to have a regular and orderly shape, and easily adapt to large-sized bone defects, but bioceramic granules can fill random bone defect cavities to meet a wider range of clinical applications (CHOI et al., 2014; Dong et al., 2022; Huang et al., 2022; Hayashi et al., 2023). Unfortunately, the osteoconductive capability of the two types of implants with similar internal pore architectures has not yet been designed for comparison. In the present study, the IWP-pore bioceramic scaffolds of 57% and 70% in porosity were 3D-printed, and meanwhile, the sintered scaffolds were compressed to granular fillers. It is interesting that the porosity and morphology of fillers with scaffold and granular morphologies could mediate the different osteogenic capacities in the early stage and during the long-term repair process.

Numerous studies have emphasized that the pore geometry provides space for osteogenesis and contributes to bone formation (Kuboki et al., 1998; Wang et al., 2022). In the fabrication of porous materials, 3D printing has gained much attention for its high accuracy and ability to produce complex bionic structures that are not possible with traditional fabrication methods (Su et al., 2022; Wei et al., 2022). Compared to conventional structures, various TPMS bionic scaffolds designed by computer-aided modeling have a high surface-to-volume ratio, greater stress dispersion, and higher permeability (Vijayavenkataraman et al., 2018). As for the TPMS pore scaffolds, it was found that the different shapes, pore sizes, and curvatures of the TPMS structures resulted in different osteoconductive and angiogenic capabilities (Li et al., 2021; Li et al., 2023; Zhang Q. et al., 2022; Wu et al., 2022; Yang et al., 2022). Moreover, the complex topology of the TPMS carrier has excellent mechanical properties (Lu et al., 2021). The addition of 3D printing helps gain insights into the influence of the structural geometric features of the pore network on the mechanical properties and stability of the TPMS scaffold itself.

The IWP pore scaffold was created from mathematical functions, with smooth interconnected interiors, zero mean curvature, and similar characteristics to bone trabeculae (Al-Ketan et al., 2018). The porous scaffold was produced with permeable internal pores, and the granules exhibited a porous structure (Figure 2A). Furthermore, the 2D/3D μCT-reconstructed images showed a well-connected pore network within all implants (Figure 2C). It was observed that the IWP pore scaffolds with the same pore size showed similar changes in the stress–strain trend but significant differences in compressive strength due to different porosities (Figure 2B). In general, when the TPMS pore scaffolds are compressed, the center layer pore wall often collapses first, leading to a stress jump in the stress–strain curves.

Up to now, more and more studies have paid attention to the relationship between pore size (in the range of 300–900 μm) and biological performances of porous materials (Vail et al., 1994; Hayashi et al., 2022). Due to the direct influence of the pore size and angiogenic efficiency, some researchers have struggled with the optimal pore dimension for achieving appreciable bone ingrowth with time (Wu et al., 2021). It is reasonable to consider that an increase in the size of the lateral opening results in more bone tissue infiltrating the internal opening of the scaffold at an earlier stage, thereby promoting more bone formation. Taniguchi et al. prepared porous titanium implants of 65% in porosity for *in vivo* studies (average pore size 300, 600, and 900 μm) and found that 600-μm pore implants had high implant fixation capacity at 2 weeks and optimal osteogenesis at 8 weeks with 600-μm pore implants (Taniguchi et al., 2016). Therefore, the expected scaffold pore size of 550 μm was selected for this study, and the actual pore sizes for the 57-S and 70-S scaffolds after sintering were 458 and 464.2 μm, respectively. The 57-G and 70-G granules had an average pore size of 316.3 and 505.8 μm, respectively. All of the implants were able to be beneficial for new bone ingrowth and vascularization (Figure 2D).

To investigate the effect of porosity on the mechanical properties of CSi-Mg5 scaffolds, this study fabricated IWP structures with two porosities by varying the pore wall thickness based on the TPMS pore network model. Despite some attempts to alter the porosity of the biomaterial by traditional pore manufacturing techniques, the changes in porosity are often accompanied by the pore size (Kasten et al., 2008). Pore size is also a key factor in the mechanical properties of the porous scaffolds. In our study, the IWP pore scaffolds with a pore size of nearly 550 μm were successfully fabricated, and the 57-S and 70-S scaffolds exhibited appropriate compressive strength (>12 MPa; Figure 2B). Meanwhile, the CSi-Mg5 scaffolds remained above 6 MPa in compressive strength after soaking in the Tris solution for 4 weeks, which was able to match the strength of trabecular bone (2–6 MPa) (Subramaniam et al., 2016). Obviously, the scaffold strength showed a decreasing trend with the increasing scaffold porosity and immersion time (Figure 3C). Notably, our previous study has demonstrated that the introduction of 3%–10% Mg in CSi could enhance the sintering densification of the bioceramics and thus significantly improve the mechanical properties (Xie et al., 2016; Wu et al., 2022; Li et al., 2023).

On the other hand, the porosity of the scaffolds plays a crucial role in influencing material degradation. The specific surface area of the granular material was higher than that of the scaffold, with −15.31 and −14.15 m^2^/kg for the model encapsulated 57-G and 70-G, respectively, and −4.85 and −4.30 m^2^/kg for the prepared 57-S and 70-S scaffolds, respectively (Table 1). This meant that the granular biomaterial was exposed to more Tris solution during the immersion experiments, while the dissolution drive caused the bioceramics to release large amounts of calcium, magnesium, and silica ions early on before leveling off (Figures 3D–F). The granule groups therefore exhibit faster biodegradation (Figure 3C). By reducing the porosity of the material, the corrosion resistance can be improved due to the reduction in the specific surface area and *vice versa*. Our work shows that CSi-Mg5 is actively degraded *in vitro* in the Tris solution, with mass loss showing a strong dependence on the specific surface area. High porosity enhances inward bone growth and osseointegration of postoperative implants, but the ideal scaffold for bone tissue repair should have an appropriate porosity and degradation rate (Karageorgiou and Kaplan, 2005; Kasten et al., 2008). In the initial stages of bone repair, the space required for inward growth of the new bone tissue and the structural stability can be maintained. In the later stages of healing, gradual degradation is possible, with the new bone tissue eventually filling the defect area completely.

In addition, it is worth mentioning that the scaffolds and granules did not induce an inflammatory response *in vivo* in the early stages (Figures 4C, 5). The effect of the porous implants on osteogenesis was investigated *in vivo* at several time points (4–16 weeks). The pores within the scaffold and the interconnected pores between the granules showed that the new bone fitted tightly into the bioceramic struts and continued to grow, confirming that bioceramics responded to osteostimulative activity *in vivo*. The superior osteogenesis of the 57-S scaffold compared to the 70-S scaffold can be attributed to the greater surface area of the scaffold resulting in the release of more ions and bone stimulation from ionolysis products in the early stages. In particular, 57-G showed more pronounced bone tissue at 4 and 10 weeks, possibly due to the smaller pore size and faster biodegradation rate in the 57-G granule fillers, resulting in superior osteoblast migration and growth.

Histological analysis also confirmed that the granule groups showed more appreciable early-stage ingrowth of the new bone tissue, whereas the scaffold group took longer for the new bone to infiltrate the entire scaffold, possibly due to the synergistic activation of ionolysis products and irregular internal pore networks stimulating the ingrowth of bone tissue, with regular and intact internal pore networks leading to longer osteoblast migration. Interestingly, the 57-G and 70-G scaffolds showed a decrease in osteogenic BV/TV at 16 weeks, while the 57-S scaffold showed the best osteogenic volume (Figure 5D). This may be due to the new bone tissue remodel after the formation of the bone scab in the granular groups and the 70-S scaffold group at 16 weeks.

Finally, it is concluded that the strength decay of CSi-Mg5 scaffolds is related to their porosity size. All CSi-Mg5 scaffolds have a compressive strength comparable to that of trabecular bone after long immersion in an aqueous medium. As for the corresponding porous granular biomaterials, the specific surface area was increased and a faster biodissolution rate was exhibited compared to the scaffold *in vitro*. Understandably, the variation in the pore structure can also alter the stability of the bioceramics *in vivo*, with the early-to middle-stag granular biomaterial facilitating bone tissue invasion throughout the implant through massive ion release, and the scaffold providing continuous mechanotransduction and osteoconduction. These studies have the potential to further elucidate the influence of the pore structural feature and morphology of the implants during osteogenesis, particularly in favor of achieving a balance between pore architecture optimization and biodegradation during the prolonged phase.

## 5 Conclusion

In summary, bioceramic scaffolds with excellent long-term mechanical stability and controlled degradation, as well as granular scaffolds, were fabricated using 3D printing technology, and the structural parameters of porosity and pore size, *in vitro* biodegradation behavior, and *in vivo* bone tissue regeneration were systematically evaluated. Magnesium-doped CSi bioceramic scaffolds with different porosities show high mechanical properties. At the same time, the differences in porosity and structure resulted in significant osteogenic differences between the scaffold and the granular filler at early and late stages. These experimental results indicate that the degradation rate and long-term mechanical support of both bioceramic scaffolds and granular implants with different degradation rates have interesting biological properties in enhancing the bone repair effect. Therefore, the study of the rational design of biomaterial structures and derived structures by 3D printing is expected to provide guidance for the selection of clinical translation and applications.

## Data Availability

The original contributions presented in the study are included in the article/Supplementary Material. Further inquiries can be directed to the corresponding authors.
