# Supplementary figures and images for: Comparison of osteogenic capability of 3D-printed bioceramic scaffolds and granules with different porosities for clinical translation
Source: Front Bioeng Biotechnol. 2023 Sep 28;11:1260639. doi: 10.3389/fbioe.2023.1260639 (PMC10569306; doi:10.3389/fbioe.2023.1260639)

**Figure 1**.XRD patterns of the CSi and CSi-Mg5 powders after calcining at 850°C.


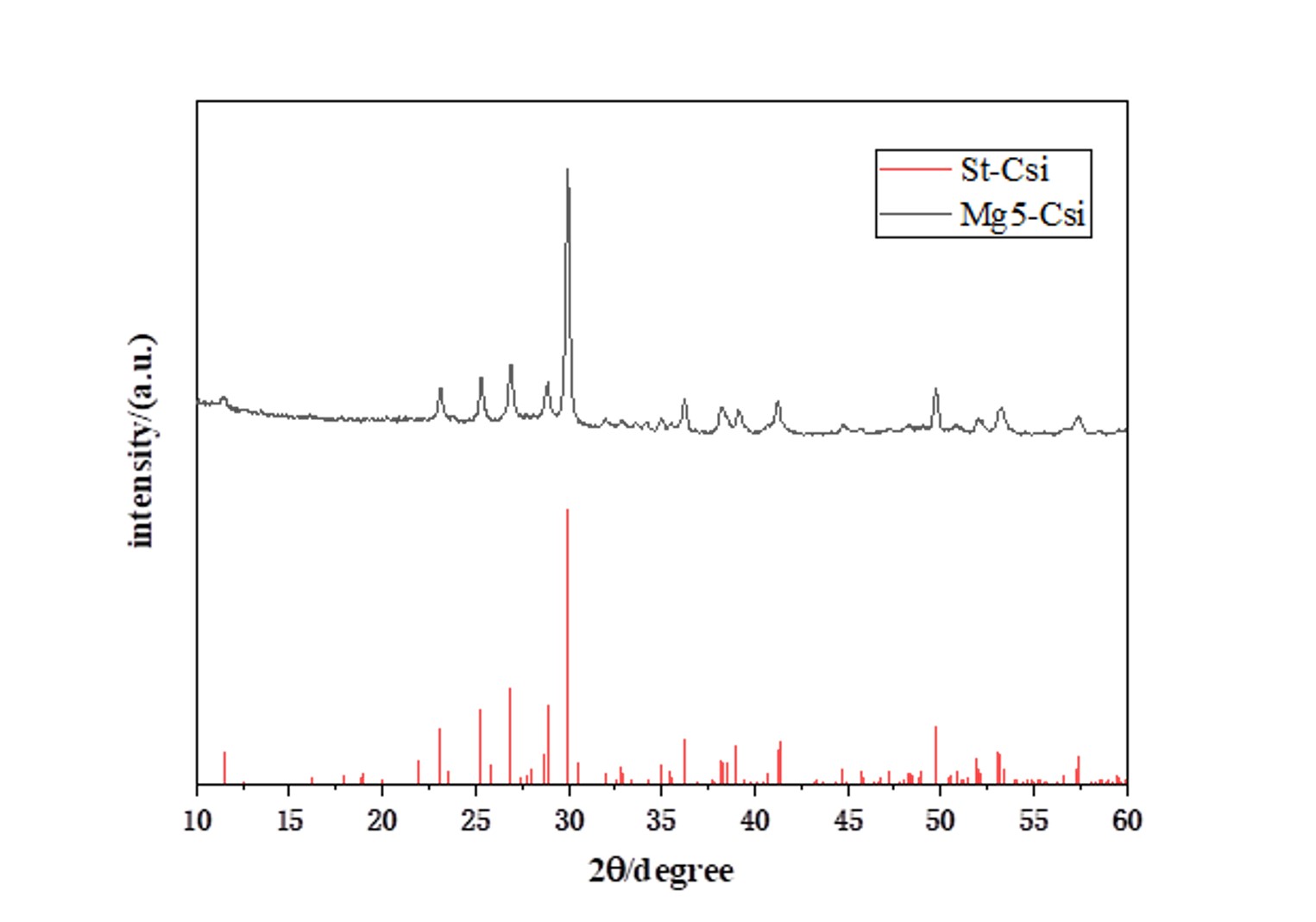

Supplement: Supplementary file 1 [file Table1.DOCX]
